# A novel three-dimensional evaluation protocol to assess the consistency of critical screw angles between preoperative planning and 3d-printed patient-specific acetabular revision prostheses

**DOI:** 10.3389/fbioe.2025.1604285

**Published:** 2025-08-18

**Authors:** Dinghao Luo, Junxiang Wu, Zhaoyang Ran, Wen Wu, Lei Wang, Kai Xie, Yongqiang Hao

**Affiliations:** ^1^Department of Orthopedics, Shanghai Ninth People’s Hospital, Shanghai Jiao Tong University School of Medicine, Shanghai, China; ^2^ Shanghai Key Laboratory of Orthopedic Prostheses, Shanghai, China; ^3^3D Printing Technology Clinical and Translational Research Center, Shanghai Ninth People’s Hospital, Shanghai Jiao Tong University School of Medicine, Shanghai, China; ^4^ Shanghai Engineering Research Center of Orthopedic Prostheses and Personalized Medical Equipment, Shanghai, China

**Keywords:** 3D pelvic coordinate system, acetabular revision prosthesis, 3D printing, customization, critical screws

## Abstract

**Background:**

Screw fixation is pivotal for prosthetic stability. For 3D-printed customized acetabular revision prostheses designed for complex, large-scale bone defects, precise adherence to preoperative screw trajectory planning is critical. However, there remains a lack of standardized three-dimensional (3D) evaluation protocols to quantify intraoperative screw angular alignment fidelity relative to preoperative digital plans, hindering universal validation criteria.

**Methods:**

A total of 11 patients were stratified into two groups based on postoperative Harris Hip Scores and severe complication rates: the better outcome group and the regular outcome group. A 3D pelvic coordinate system was established using anatomical landmarks. Two biomechanically critical screws per prosthesis were selected to quantify 3D angular deviations between postoperative and preoperative plans. The efficacy of this method was compared with simulated anteroposterior radiograph-based 2D measurements. Inter- and intra-observer consistency (kappa statistics) were evaluated to assess reproducibility.

**Results:**

High inter-observer (κ = 0.88) and intra-observer (κ = 0.87, 0.75) agreement confirmed method reliability. While individual critical screw deviations showed no significant intergroup differences, the cumulative angular deviation of critical screws was significantly lower in the better outcome group (p = 0.0147). In contrast, 2D radiographic analysis failed to distinguish intergroup differences in cumulative deviations (p = 0.1489), demonstrating reduced clinical relevance.

**Conclusion:**

This 3D assessment protocol robustly correlates with clinical outcomes, providing a validated tool for evaluating preoperative-to-postoperative fidelity in customized acetabular revision prostheses. To our knowledge, this is the first CT-based 3D coordinate system study quantifying critical screw alignment accuracy in patient-specific prostheses, with clinical validation.

## Graphical Abstract

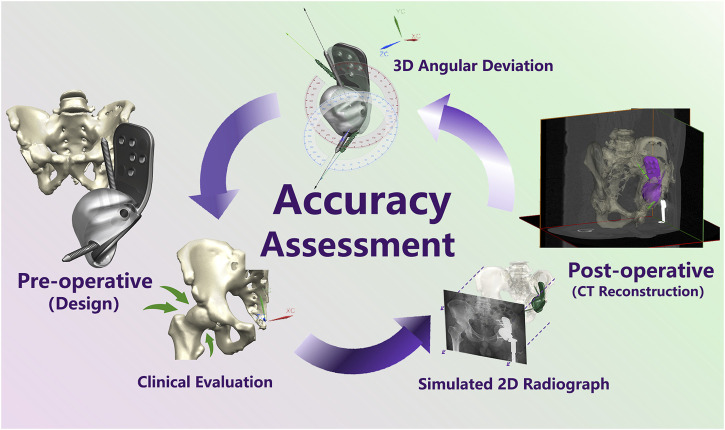

## 1 Introduction

Total hip arthroplasty (THA) has been consistently regarded as one of the most successful surgical innovations of the 20th century. However, the increasing prevalence of implant failures due to aseptic loosening, periprosthetic fractures, and infections has led to a progressive rise in revision arthroplasty procedures (Velasquez Garcia et al., 2024; Brachet et al., 2023). Despite the application of a large number of new technologies such as bioprinting and coating in this crucial field (Zhan et al., 2025; Fa-Binefa et al., 2025; Ding et al., 2020), compared to primary THA, revision surgeries present greater technical complexity, with reported complication rates reaching 40% in certain studies (Andrzejewski et al., 2023; Zampelis and Flivik, 2021; Patel et al., 2015). Furthermore, the substantial incidence of secondary revisions due to failed revision prostheses underscores both the clinical challenges and the urgent need for improved outcomes in this domain (Hasegawa et al., 2017).

Among factors ensuring long-term stability of acetabular revision prostheses, screw fixation plays an indispensable role. Screws mechanically integrate the prosthesis with acetabular bone through axial compression and angular resistance to shear stresses. In recent years, research related to orthopedic implants and screws has attracted considerable attention, particularly in the field of biomechanics, where substantial progress has been made. Notable studies have focused on different acetabular fracture fixation strategies, the selection of appropriate boundary conditions, and the analysis of stress and displacement distributions in both bone and implant structures. However, despite these advances, studies specifically addressing the accuracy of screw placement—particularly in the context of screws used in 3D-printed, patient-specific implants—remain scarce (Hao et al., 2011; García et al., 2000; Khajavi et al., 2010; Karpiński et al., 2016; Zubrzycki et al., 2018). For 3D-printed patient-specific acetabular revision prostheses targeting complex bone defects, screw trajectory accuracy assumes heightened significance. These prostheses inherently exhibit reduced bone-implant contact compared to primary THA components, with limited interfacial congruence even after personalized design (Schumann et al., 2015; Palit et al., 2022; Li et al., 2019; Cao et al., 2019). Consequently, screw-derived mechanical support becomes the principal determinant of prosthetic stability. The trajectories of the longest, biomechanically critical screws are meticulously planned preoperatively, accounting for residual bone stock quality, stress distribution patterns, and load-bearing axes. Precise intraoperative execution ensures optimal screw placement within mechanically competent bone regions, facilitating proper stress transfer along the trunk-pelvis-screw-acetabular cup-femoral stem-lower limb axis (Zampelis and Flivik, 2021; Bayraktar et al., 2017). Conversely, angular deviations may predispose to stress shielding, screw loosening, periprosthetic fractures, and ultimately aseptic failure, particularly when affecting biomechanically critical screws (Palit et al., 2022).

Objective assessment of screw placement accuracy thus serves dual purposes: prognostic stratification and iterative refinement of prosthetic design/surgical protocols (Li et al., 2019; Henckel et al., 2023; Sugano et al., 2012). Conventional radiographic methods remain the clinical mainstay for evaluating standardized prostheses (Bayraktar et al., 2017), yet suffer from projection artifacts, anatomical superimposition, and reduced accuracy in settings of acetabular bone loss/remodeling (Schumann et al., 2015). Although CT-based 3D reconstructions overcome many 2D limitations, existing protocols exhibit poor adaptability to patient-specific prostheses and significant operator-dependent variability (Lu et al., 2023). In addition, although some exploratory studies have investigated the accuracy of screw implantation in 3D-printed, patient-specific implants and have attempted to optimize surgical techniques and evaluate outcomes, the assessment of screw angulation remains limited. Moreover, these studies lack detailed three-dimensional evaluations, which considerably restricts their applicability and reference value for improving personalized implant design in this context (Eraly et al., 2016).

This context necessitates an ideal CT-based 3D evaluation protocol that: 1) prioritizes biomechanically critical screws over exhaustive analyses, ensuring clinical practicality while outperforming 2D methods; 2) accommodates highly individualized preoperative plans for 3D-printed prostheses while maintaining clinical correlation; and 3) demonstrates strong reproducibility across operators and measurement sessions. Addressing these requirements, we propose a novel 3D pelvic coordinate system-based methodology for quantifying preoperative-postoperative consistency of critical screw placement in customized acetabular revision prostheses. This study systematically validates the protocol’s observer consistency, clinical relevance, and superiority over 2D radiographic simulations. To the best of our knowledge, this represents the first such methodology in the literature, with potential to revolutionize precision assessment in patient-specific acetabular reconstruction.

## 2 Materials and methods

### 2.1 Patients and grouping

**FIGURE 1:**
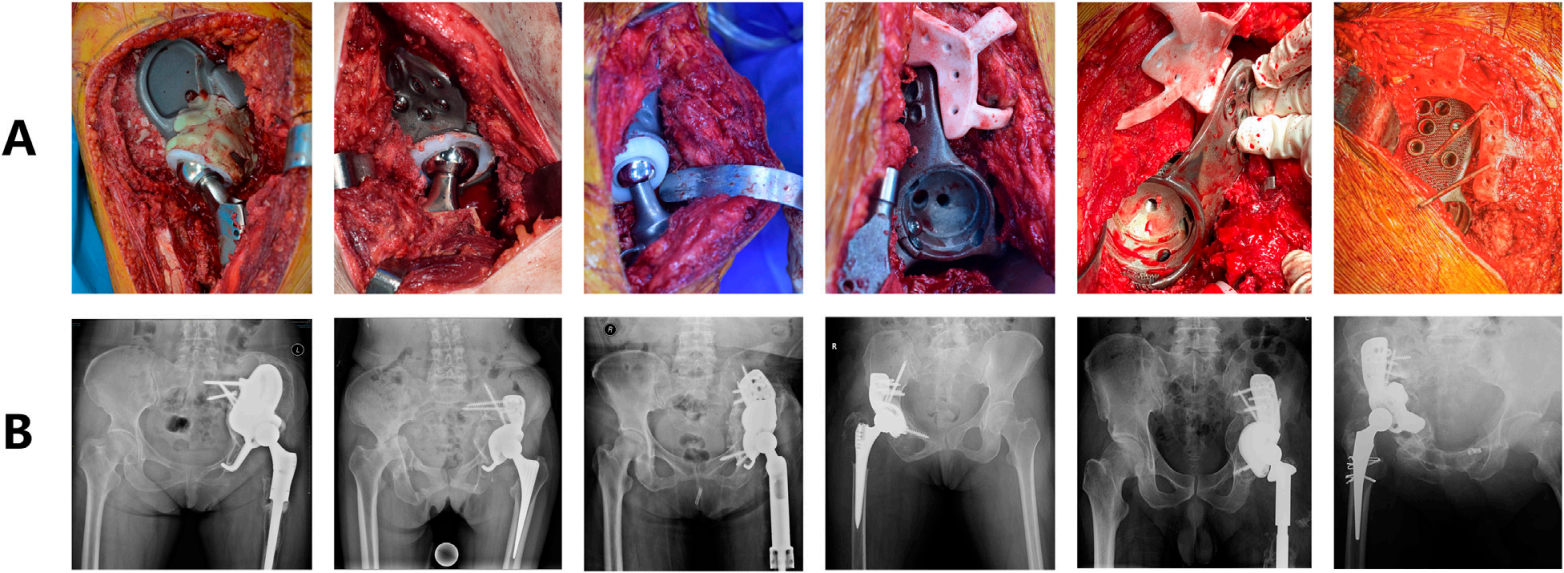
Representative intraoperative surgical field and corresponding postoperative plain radiographs of selected patients. **(A)** Intraoperative photograph showing prosthesis implantation. **(B)** Postoperative anteroposterior pelvic radiograph of the patient.

Between 2014 and 2021, our research team conducted hip revision arthroplasty using 3D-printed patient-specific prostheses in 11 patients (Figure 1), with a mean follow-up duration of 47.9 months (range: 17–98 months). Postoperative evaluations included Harris Hip Scores (HHS) and pelvic CT scans, with meticulous documentation of severe complications: joint dislocation, prosthesis removal due to fracture/infection, and permanent nerve palsy. Patients achieving HHS ratings of “good” or better (≥80 points) without these complications were classified into the “better-outcome group,” while others constituted the “regular-outcome group.”

This study received ethical approval from Shanghai Jiao Tong University School of Medicine (SH9H-2014-54), conducted in strict compliance with the Declaration of Helsinki (JBJS 79A:1089-98, 1997). Patient confidentiality was maintained according to Health Insurance Portability and Accountability Act (HIPAA) regulations. All prostheses and bone screws implanted in the patients included in this study were sourced from the same manufacturer (Shanghai Shengshi MedTech Co., Ltd., China) and were all made of Ti6Al4V titanium alloy.

### 2.2 Data processing of pelvic anatomy, prostheses, and critical screw structures

**FIGURE 2:**
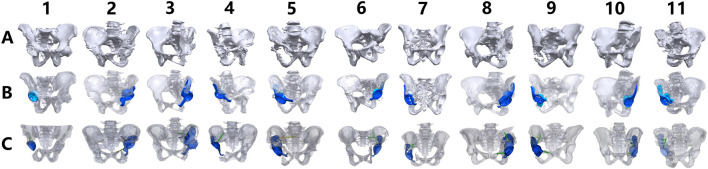
Preoperative planning and postoperative CT volumetric reconstructions across all cases. **(A,B)** Preoperative digital models displaying pelvic osseous structures (grayscale volumetric renderings) alongside prosthetic components (yellow: acetabular cup; red: fixation screws). **(C)** Postoperative CT-derived 3D reconstructions illustrating bone-prosthesis integration, with osseous structures rendered in grayscale and prosthetic components in yellow.

All structural analyses were performed using both preoperative planning data (prosthesis design files) and postoperative CT-based 3D reconstructions for comparative evaluation (Figure 2).

During preoperative planning, prosthesis design files were analyzed in UG NX 12.0 (Siemens AG, Germany) to visualize pelvic bone structures, prosthetic bodies, and all screws. Based on a combination of clinical experience, biomechanical principles, and interdisciplinary medical-engineering design, two biomechanically critical screws were selected for each prosthesis using stringent criteria:

1. For prostheses without pubic screw fixation, the two longest screws oriented along the primary load-bearing axis toward the sacroiliac joint, positioned at the highest anatomical location, were selected.
2. For prostheses with pubic screw fixation, one pubic screw and the longest sacroiliac-oriented screw were selected as the critical screws.

In each case, the two critical screws were initially selected by a physician with extensive experience in interdisciplinary medical-engineering collaboration. The selection was then independently reviewed by two senior physicians with similarly extensive experience. Only when all three selections were completely consistent were the two critical screws definitively confirmed, thereby ensuring the rigor and reliability of the selection process. To ensure the accuracy and consistency of imaging data and subsequent research outcomes in this study, all key personnel involved in the aforementioned processes—including implant designers, structural analysts, surgical planning personnel, and surgical operators—remained consistent throughout the study period. This approach facilitates the long-term development of relevant technical expertise and promotes positive, cumulative experience within the research team.

Postoperative pelvic CT data were reconstructed using Mimics Medical 20.0 (Materialise, Belgium) to reproduce the spatial relationships of pelvic bones, prosthetic bodies, and critical screws. These reconstructions were exported as STL files and co-registered with preoperative models in UG NX 12.0 for comparative analysis.

#### 2.2.1 Finite element analysis to differentiate surgical vs. design factors in acetabular revision outcomes

To determine whether observed intergroup therapeutic disparities stemmed from inherent screw design quality differences rather than surgical execution variability, we conducted finite element mechanical analysis to confirm equivalent intrinsic screw design quality between groups. This methodological approach substantiates that outcome discrepancies primarily arose from intraoperative installation quality rather than fundamental design characteristics.

The finite element model incorporated prosthesis geometry with screw holes derived from 3D structural blueprints. A detailed pelvic model specified:

Specifically, the STL files of the bony structures were processed using Geomagic Studio 2014 (Geomagic, United States) for reverse modeling. Following surface patch division, mesh generation, and surface fitting, the models were converted into solid entities and imported into UG NX 12.0 in IGES format. Cortical and cancellous bone regions were defined, with the cortical thickness set to 2 mm. Subsequently, the three-dimensional models of the prosthesis and the solid bony structures were exported in X-T format and imported into Ansys Workbench 19.2 for FE analysis.

The material properties assigned to each component are detailed in Table 1, with all materials assumed to be homogeneous and isotropic. Bonded contact conditions were applied at all bone–prosthesis–screw interfaces. A fixed support constraint was applied to the inner surface of the acetabular cup, and a vertical downward load of 650 N was applied to the bone–prosthesis and screw–prosthesis interfaces to simulate physiological loading.

**TABLE 1:** Material properties of bones and prosthesis components in FE models.

Component	Material	Young’s modulus (Pa)	Poisson’s ratio
Pelvic cortical bone	Cortical bone	1.7 × 10^10^	0.3
Pelvic trabecular bone	Trabecular bone	1.5 × 10^8^	0.2
Prosthesis	Ti6Al4V	1.1 × 10^11^	0.3
Bone screws	Ti6Al4V	1.1 × 10^11^	0.3

A representative case was selected for mesh generation using the automatic meshing algorithm. To ensure mesh quality, systematic evaluation of element regularity and localized remeshing were implemented to minimize stress concentration artifacts. Mesh independence was confirmed: when the mesh resolution was set to 7, the model contained 380,575 nodes and 239,003 elements, and the maximum equivalent stress on the prosthesis surface varied by less than 5% compared to resolutions of 5 and 6, indicating convergence. Therefore, a mesh size corresponding to an average element area of 8.53 × 10^−5^ m^2^ was adopted.

The equivalent stress and maximum principal stress of the FE model were calculated. Model validation was performed based on the workflow proposed by Liu et al. (2016) and Liu et al. (2019), including comparative analysis of displacement patterns between the current and previously published models and benchmarking of stress distributions against established standards in computational biomechanics.

The distribution and magnitude of displacement and stress observed in our previous studies demonstrated substantial agreement with the findings reported by Hua et al., thereby confirming model validity (Liu et al., 2016; Liu et al., 2019). After confirming the validity of the modeling approach, FE models for all other cases were constructed using the same protocol. Bonded contact conditions were applied at all bone–prosthesis, prosthesis–screw, and screw–bone interfaces. The inner surface of the acetabulum was constrained with fixed support, and a vertical downward load of 650 N was applied to the bone–prosthesis contact surface. Equivalent stress and maximum principal stress distributions were subsequently calculated for all models.

### 2.3 Establishment of standardized 3D pelvic coordinate system

**FIGURE 3:**
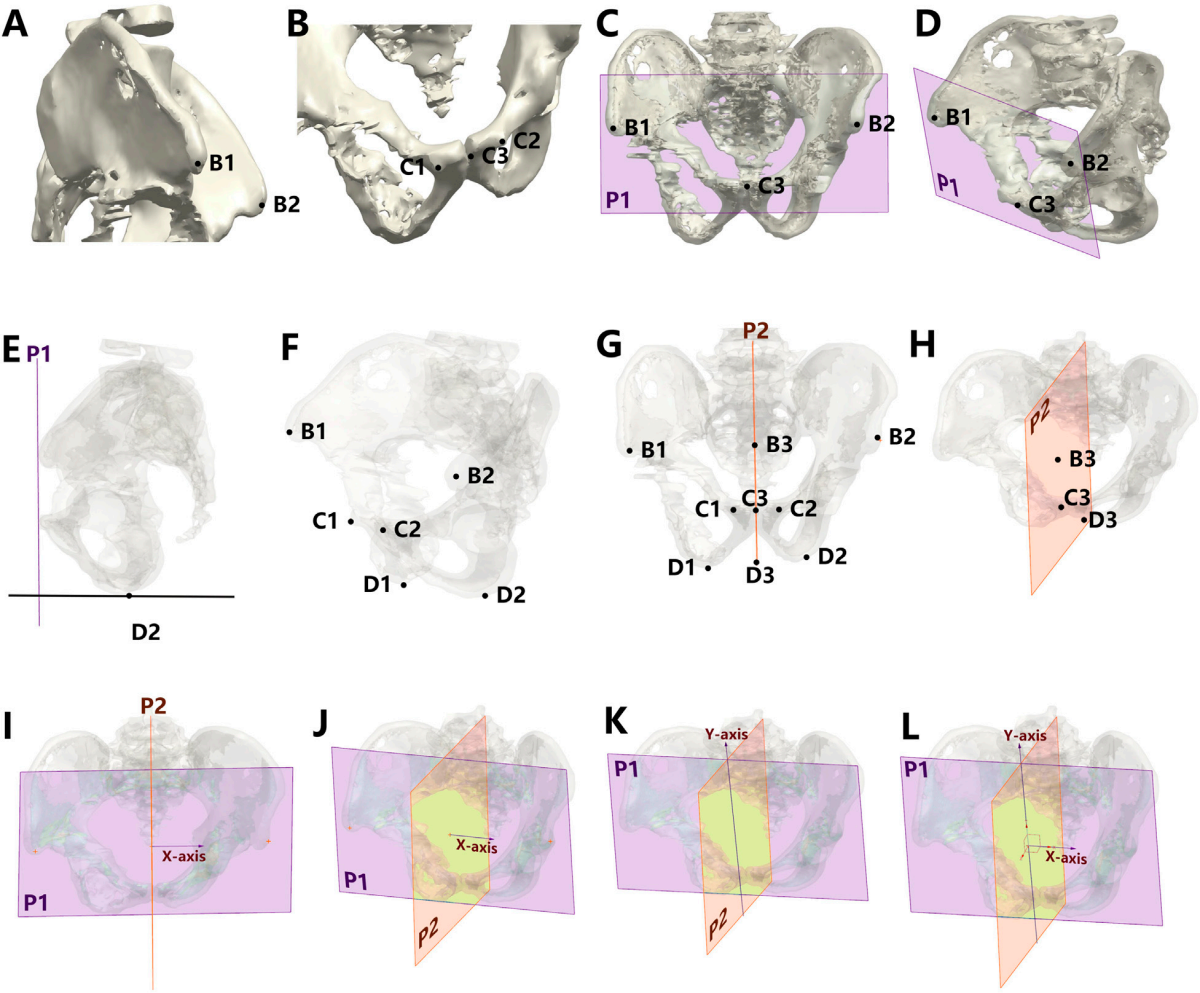
Protocol for establishing the standardized 3D pelvic coordinate system. **(A)** Bilateral anterior superior iliac spine (ASIS) landmark identification (B1-B2). **(B)** Pubic tubercle midpoint determination (C1-C2: bilateral tubercles; C3: midpoint). **(C,D)** Frontal plane construction (blue plane P1) triangulating ASIS points (B1-B2) and pubic midpoint (C3). **(E)** Objective localization of ischial tuberosity inferior points (D1-D2) using elevation vectors perpendicular to P1. **(F–H)** Midsagittal plane derivation (green plane P2) from midpoint triad: ASIS midpoint (B3), pubic midpoint (C3), and ischial midpoint (D3). **(I,J)** Coordinate system origin (B3: ASIS midpoint) and X-axis definition (perpendicular to P2). **(K,L)** Full coordinate system establishment: Y-axis as P1-P2 intersection line (craniocaudal orientation), Z-axis orthogonal to X/Y-axes, completing anatomical reference framework.

As detailed in Figure 3, the pelvic coordinate system was constructed in UG NX 12.0:

• **Coronal plane:** Defined by the midpoint between bilateral anterior superior iliac spine (ASIS) and pubic tubercles (Fischer et al., 2019)
• **Midsagittal plane:** Established using midpoints of ASIS, pubic tubercles, and bilateral ischial tuberosity inferior points
• **Coordinate origin:** ASIS midpoint
• **Axial definitions:** Y-axis (vertical)-----Intersection line of coronal planes; X-axis (horizontal)-----Perpendicular to midsagittal plane through origin; Z-axis (sagittal)-----Orthogonal completion of right-handed system.

Identical coordinate systems were independently created for preoperative plans and postoperative CT reconstructions.

### 2.4 Quantification of critical screw angular deviation

For each critical screw:

1. Two concentric circles were generated around screw head and tail regions in their respective coordinate systems
2. Central axes were established by connecting circle centroids
3. Three-dimensional coordinates of axis endpoints were recorded
4. Spatial angles between preoperative and postoperative screw axes were calculated using vector analysis of endpoint coordinates

The cumulative angular deviation for both critical screws per prosthesis was calculated as the arithmetic sum of individual deviations.

### 2.5 Assessment of inter- and intra-observer agreement

To evaluate methodological reproducibility, two blinded biomedical engineers/physicians independently performed: coordinate system establishment; critical screw axis identification; angular deviation calculations.

This process was repeated after a 2-month interval. Observer agreement was analyzed through:

• **Intra-observer consistency:** Comparison of each evaluator’s two-round measurements
• **Inter-observer consistency:** Comparison of second-round results between evaluators

Angular deviations were categorized into four grades: Grade A: 0° ≤ deviation < 10°; Grade B: 10° ≤ deviation < 15°; Grade C: 15° ≤ deviation < 20°; Grade D: ≥20°

Cohen’s kappa (κ) statistics were employed for agreement analysis with established thresholds: κ < 0.40: poor agreement; 0.40 ≤ κ ≤ 0.75: moderate agreement; κ > 0.75: excellent agreement.

### 2.6 Comparative analysis between CT-Based 3D coordinate system method and simulated radiographic 2D measurement

To simulate conventional anteroposterior radiographic measurements:

1. Screw axes were projected onto the XY (coronal) plane
2. Projected endpoints were recorded
3. Angular deviations were recalculated in 2D space using projected coordinates

The clinical relevance of both 3D and simulated 2D methods was evaluated through their ability to discriminate between outcome groups.

### 2.7 Statistical analysis

SPSS Statistics 27.0 (IBM, United States) was utilized for all analyses. For critical screw deviation comparisons, normality was assessed using Shapiro-Wilk test, variance homogeneity was evaluated through F-test. Group comparisons employed Mann-Whitney U test for non-normal distributions, independent t-test for normal distributions with homogeneous variances, and Welch’s t-test for normal distributions with heterogeneous variances. Categorical agreement was analyzed using Cohen’s κ with chi-square testing. Statistical significance was set at p < 0.05.

## 3 Results

### 3.1 Consistent design quality across prosthesis groups and screw holes in mechanical validation

**FIGURE 4:**
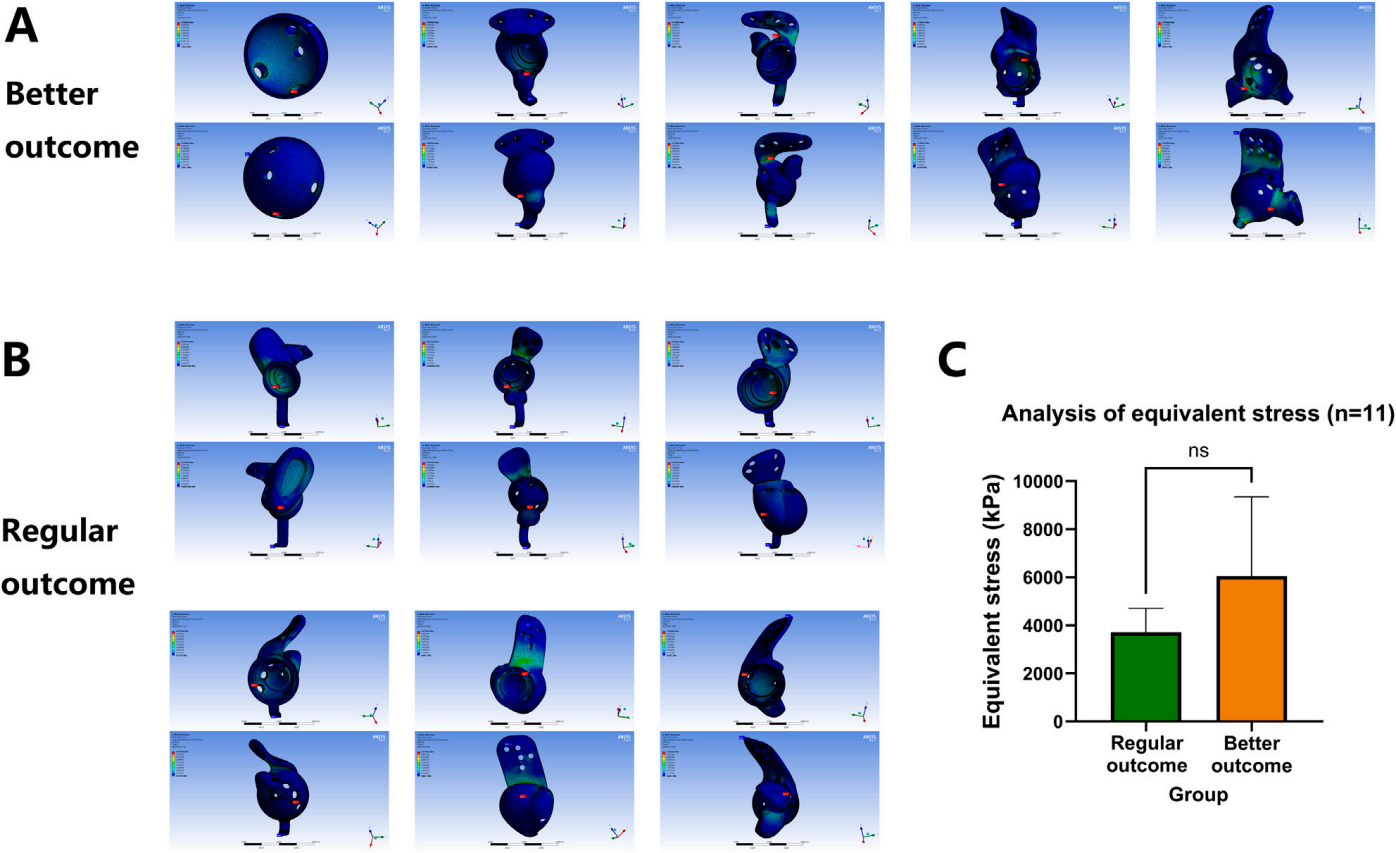
Finite element equivalent stress consistency analysis of prosthesis and screw-hole structural design quality between groups. **(A,B)** Equivalent stress calculation across prosthesis groups and screw holes. **(C)** Comparative analysis of equivalent stress distributions.

**FIGURE 5:**
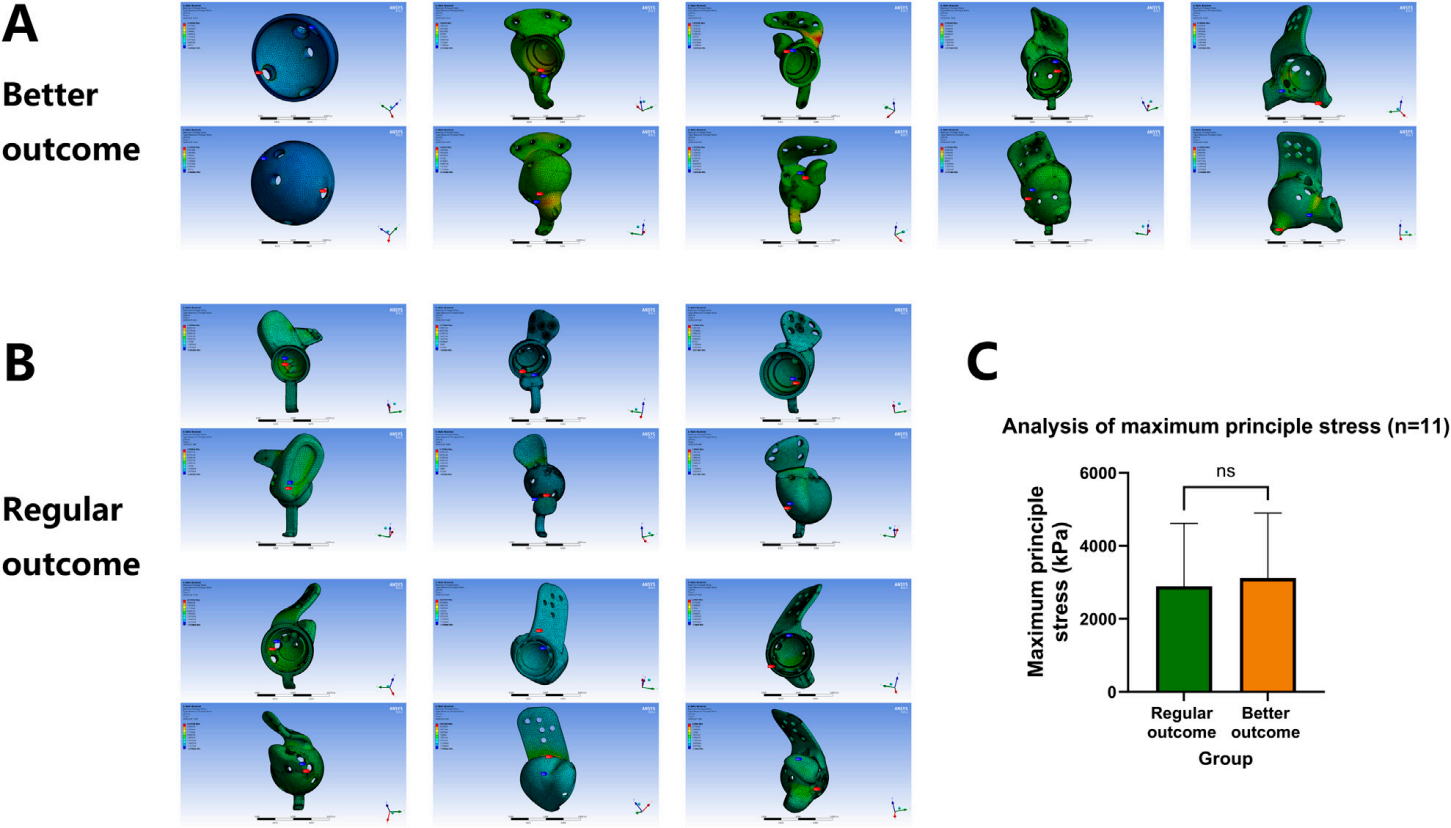
Finite element maximum principal stress consistency analysis of prosthesis and screw-hole structural design quality between groups. **(A,B)** Maximum principal stress calculation across prosthesis groups and screw holes. **(C)** Comparative analysis of maximum principal stress distributions.

As shown in Figures 4, 5, the equivalent stress and maximum principal stress distributions of both prosthesis groups were calculated. Finite element simulation analysis revealed no significant differences in equivalent stress (6,054 ± 3,296 kPa VS 3,709 ± 1,003 kPa, p = 0.1906) or maximum principal stress (3,118 ± 1,778 kPa VS 2,891 ± 1,727 kPa, p = 0.8363) between groups (Figures 4, 5; Table 2) after comprehensive consideration of screw functions in prosthesis fixation and stress transmission. These findings demonstrate comparable design quality between prosthesis groups and screw holes under original design parameters, indicating no design flaws that would induce abnormal stress distribution patterns, compromise prosthesis stability, or negatively impact clinical efficacy through suboptimal screw-hole configurations.

**TABLE 2:** Statistical analysis of prosthesis stress (n = 11).

Parameter	Better outcome group (n = 5)	Regular outcome group (n = 6)	T value	P
Equivalent stress (kPa)	6,054 ± 3,296	3,709 ± 1,003	1.533	0.1906
Maximum principal stress (kPa)	3,118 ± 1778	2,891 ± 1727	0.213	0.8363

### 3.2 Excellent inter- and intra-observer consistency in 3D angular deviation measurement

The critical screw angular deviation grading results from two independent evaluators across two assessment rounds are presented in Table 3. Statistical analysis (Table 4) revealed substantial intra-observer consistency with κ values of 0.87 (Observer 1) and 0.75 (Observer 2). Inter-observer agreement reached an excellent κ value of 0.88. These findings confirm that our 3D angular deviation quantification method demonstrates remarkable reproducibility and reliability for clinical application.

**TABLE 3:** Interobserver grading consistency of cumulative angular deviations for critical screws across two sequential assessment rounds (n = 11).

Case	1	2	3	4	5	6	7	8	9	10	11
Observer 1 - Ⅰ	A	A	B	C	A	D	D	B	A	D	D
Observer 1 - Ⅱ	A	A	B	C	A	D	D	B	A	C	D
Observer 2 - Ⅰ	A	A	B	D	A	D	D	B	A	C	D
Observer 2 - Ⅱ	A	A	C	C	A	D	D	B	A	C	D

**TABLE 4:** Statistical analysis of inter-observer and intra-observer consistency (n = 11).

Parameter	Kappa	Consistent slices	Consistent rate
Intra-observer consistency 1	0.874	10	91%
Intra-observer consistency 2	0.750	9	82%
Inter-observer consistency	0.875	10	91%

### 3.3 Strong correlation between 3D assessment outcomes and clinical performance

**FIGURE 6:**
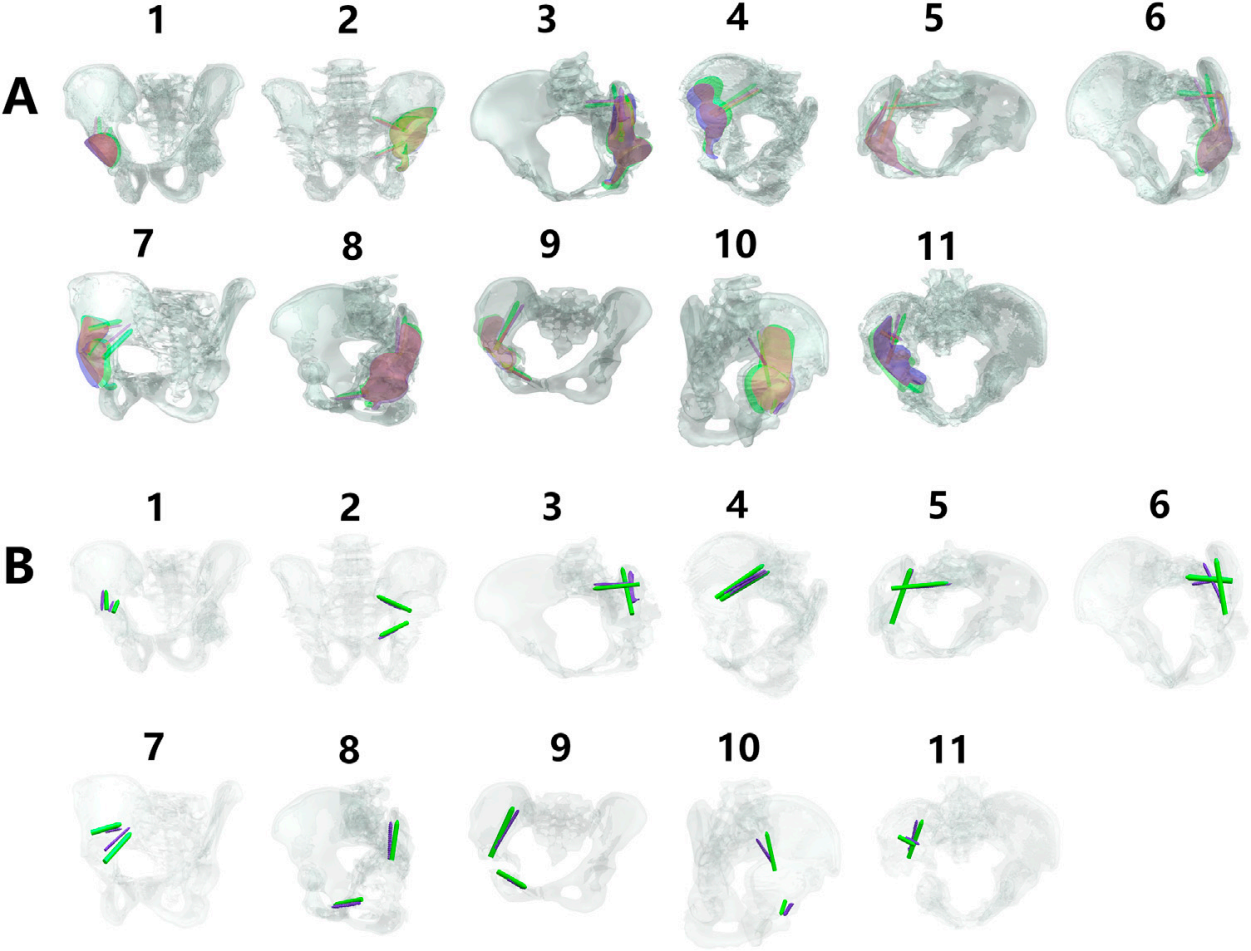
Three-dimensional angular deviation of biomechanically critical screws. **(A,B)** Comparative visualization of preoperative planning (yellow prostheses/screws) versus postoperative CT reconstructions (purple prostheses/screws) against pelvic bone renderings (grayscale).

**FIGURE 7:**
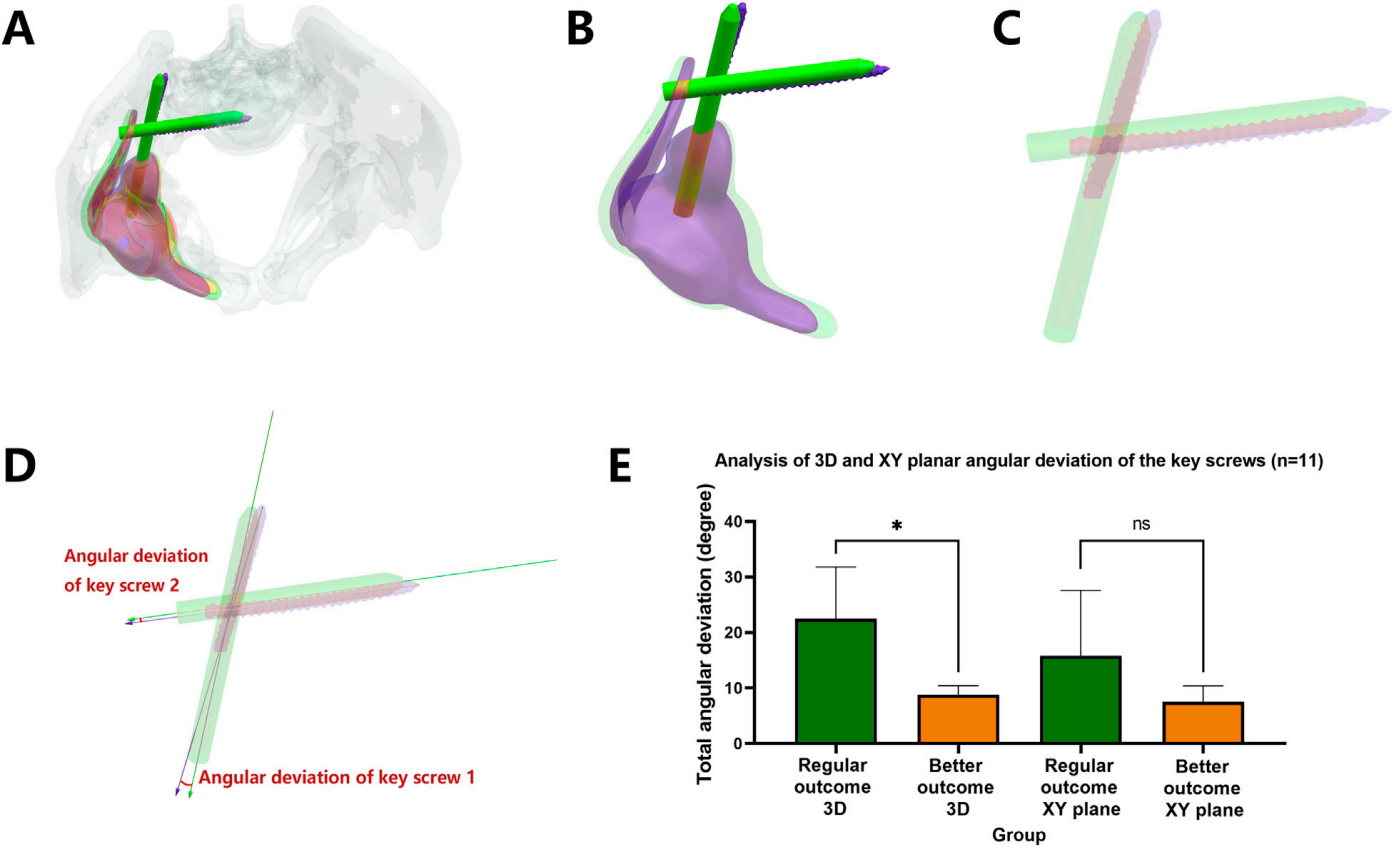
Three-dimensional angular deviation quantification for biomechanically critical screws. **(A–D)** Screw axis alignment protocol: Isolated screw structures with long-axis vectorization (yellow: planned; purple: postoperative), angular deviation was calculated. **(E)** Comparative boxplots demonstrating significantly reduced 3D angular deviations in the optimal-outcome cohort versus controls (p = 0.0147). Note: Projection onto XY plane (simulating AP radiography) eliminated intergroup differences (p = 0.1489), underscoring 3D methodology’s superior spatial resolution versus conventional 2D radiographic approximation.

As detailed in Table 5 and Figures 6, 7, the total 3D angular deviations for prostheses in the superior-outcome group averaged 8.8262° (SD ± 1.12°), significantly lower than the 22.5125° (SD ± 2.34°) observed in the standard-outcome group (p = 0.0147). Despite both groups showing normal distributions (superior group: p = 0.3083; regular outcome group: p = 0.5947), significant variance heterogeneity (F-test p = 0.0045) necessitated Welch’s t-test. This pronounced discrepancy in screw placement accuracy strongly correlates with clinical outcomes, validating the clinical relevance of our 3D assessment protocol.

**TABLE 5:** Statistical analysis of critical screw angular deviation (n = 11).

Parameter	Better outcome group (n = 5)	Regular outcome group (n = 6)	T value	P
3D critical Screw 1 angular deviation	6.5115 ± 3.0397	12.6410 ± 6.6182	−2.0266	0.0808
3D critical Screw 2 angular deviation	2.3147 ± 1.7787	9.8716 ± 4.7429	−3.3469	0.0086
Total 3D critical Screw angular deviation	8.8262 ± 1.5934	22.5125 ± 9.2867	−3.548	0.0147

### 3.4 Superior diagnostic efficacy of 3D methodology over simulated 2D radiographic approach

Table 6 and Figure 7E present the results from simulated anteroposterior radiographic measurements. While the 3D method demonstrated strong clinical correlation (Table 5), the 2D projection method showed markedly reduced discriminatory power. The total angular deviations between groups were 19.1° ± 3.2° (better-outcome) versus 23.7° ± 4.1° (regular-outcome), failing to reach statistical significance (p = 0.1489). This 54.3% reduction in intergroup discrimination efficacy (from p = 0.0147 to p = 0.1489) underscores the critical limitations of conventional 2D radiographic assessment in evaluating screw positioning accuracy.

**TABLE 6:** Statistical analysis of prosthesis and critical Screw angular deviation by simulated traditional X-ray radiography (n = 11).

Parameter	Better outcome group (n = 5)	Regular outcome group (n = 6)	T value	P
Critical Screw 1 angular deviation on XY plane	6.5578 ± 3.2277	11.0887 ± 8.5964	−1.1070	0.2970
Critical Screw 2 angular deviation on XY plane	0.9636 ± 0.6768	4.7318 ± 5.0761	−1.6332	0.1369
Total critical Screw angular deviation on XY plane	7.5215 ± 2.8632	15.8205 ± 11.7752	−1.668	0.1489

## 4 Discussion

Three-dimensional CT-based angular measurement demonstrates distinct advantages over conventional radiographic approaches, yet current research predominantly focuses on acetabular components rather than fixation screws. Study revealed that CT-based 3D reconstruction of hip prosthesis positioning showed closer alignment with robotic reference standards compared to 2D CT measurements (Henckel et al., 2023). Another investigation demonstrated the inferior reliability of radiographic acetabular angle assessments relative to CT-based 3D analysis (Davda et al., 2015). While some studies attempt to mitigate projection errors by substituting elliptical acetabular margins with prosthesis projection areas in radiographic assessments (Murphy et al., 2019), their precision remains substantially inferior to CT-based 3D reconstruction. Notably, despite recognized advantages of current CT-based 3D evaluation methods over radiography for general hip prostheses (D’Isidoro et al., 2021; Hidaka et al., 2022; Tsutsui et al., 2017), three critical limitations impede their direct application to 3D-printed patient-specific revision screws (Liang et al., 2020): 1) rigid evaluation frameworks with inadequate adaptability to customized implants, 2) poor robustness in extensive periacetabular bone defects, and 3) neglect of biomechanically critical screws in personalized revision systems.

The first limitation manifests in the inherent patient-specificity of 3D-printed screws, which incorporate residual bone morphology, implant avoidance requirements, and biomechanical considerations. This complexity renders conventional CT evaluation metrics (e.g., safe zones, standard anteversion/abduction angles) inapplicable. Such uncertainty represents a key reason why existing studies on the spatial evaluation of prosthesis positioning are difficult to directly apply to the field of screw placement in 3D-printed, patient-specific acetabular revision implants. It also highlights a critical gap in the current research landscape that urgently needs to be addressed (Zampelis and Flivik, 2021; Bayraktar et al., 2017). Our methodology circumvents this through preoperative-planning-aligned 3D deviation analysis, avoiding the postoperative-only focus of traditional CT methods. The superior screw alignment observed in our better-outcome group (vs. Regular-outcome group) validates the predictive value of preoperative-postoperative alignment algorithms, with improved clinical outcomes correlating with better angular concordance (p < 0.05).

The second challenge arises in cases with extensive periacetabular defects. Most CT-based methods rely on standardized positioning and intact pelvic anatomy, becoming error-prone when encountering severe bone loss or iliac deformities (Schumann et al., 2015; Davda et al., 2015). The widely used anterior pelvic plane (APP) coordinate system (Palit et al., 2022; Fischer et al., 2019; Yang et al., 2022), dependent on bilateral anterior superior iliac spine (ASIS), proves particularly vulnerable in iliac deformity cases. Previous studies (Bayraktar et al., 2017; Yamada et al., 2018) report declining accuracy with increasing periacetabular abnormalities. Our solution incorporates six pelvic landmarks (including bilateral ischial tuberosity inferior points) to establish a deformation-resistant X-axis. Unlike ASIS/iliac crest landmarks along the lumbar-sacral-iliac load pathway (Andrzejewski et al., 2023), ischial references maintain coordinate consistency under biomechanical stresses, enhancing measurement reliability. While study (Durand-Hill et al., 2020) improved robustness using posterior superior iliac spines, our preference for ischial landmarks reduces iliac dependence.

Thirdly, the prevailing neglect of biomechanically critical screws in current CT-based evaluations (Bayraktar et al., 2017; Davda et al., 2015) stems from their focus on standard prostheses with uniform screw orientations perpendicular to acetabular surfaces (Durand-Hill et al., 2020; Chang et al., 2021; Zhang et al., 2022). This approach proves inadequate for customized revision screws designed around complex bone defects. Our analysis of two critical screws demonstrates both clinical relevance (superior alignment correlating with better outcomes, p = 0.0147) and practical efficiency. Critical deviations from preoperative plans may induce stress concentration and fixation failure risks, underscoring the necessity for targeted evaluation.

Methodologically, while volumetric analysis enhances positional accuracy for bulk structures (Yamada et al., 2018; Nodzo et al., 2018; Iwana et al., 2013), its limited sensitivity to slender cylindrical screws (where minimal angular changes cause >90% volumetric mismatch) validates our line-based measurement approach. The significant diagnostic discordance between our 3D method and simulated 2D projections (p = 0.0147 vs. p = 0.1489) underscores the spatial information loss inherent in radiographic approximations, confirming 3D methodology’s superiority. In current clinical practice, the evaluation of prosthetic screw positioning still largely relies on conventional X-ray imaging, particularly intraoperative radiographs obtained using C-arm fluoroscopy. Under the practical constraints of the surgical setting, this remains the most comprehensive and feasible option available. However, based on the principles of three-dimensional coordinate system construction and the comparative analyses conducted in this study, it is evident that, in terms of spatial assessment—particularly angular measurement—the CT-based three-dimensional evaluation system offers fundamental advantages over conventional X-ray methods, provided that medical costs are not considered. It is worth noting, however, that through technical improvements to X-ray-based assessment methods, such as the introduction of biplanar or even multiplanar radiographic techniques, the accuracy gap between X-ray imaging and CT-based three-dimensional evaluation can be narrowed to a clinically acceptable range. This represents a promising development pathway that balances both effectiveness and cost-efficiency.

The standardized three-dimensional pelvic coordinate system proposed in this study demonstrates significant potential for broader application. In principle, as long as at least three relatively stable anatomical landmarks with low variability and minimal influence from bone defects or deformities can be identified, a similar approach can be used to establish a standardized spatial reference coordinate system. Notably, this process does not require anatomical symmetry or consideration of whether the procedure is staged. In fact, we have already applied this method in other studies involving spatial analysis of various types of bone defect implants at unilateral anatomical sites, including the femur, phalanges, and humerus. These analyses covered parameters such as screw angulation, implant positioning, and volumetric overlap ratios. However, due to the rarity of such complex and challenging cases, the sample sizes in these studies have also been relatively small, limiting the ability to definitively assess the method’s overall applicability. Moreover, we have not yet applied this approach in staged revision surgeries. Nevertheless, through future investigations, the universality and clinical value of this method are expected to be progressively elucidated.

This study has limitations including modest sample size reflecting the novel nature of 3D-printed revision prostheses, single-center design, and coronally projected simulations differing from true AP radiographs. The small sample size may serve as a potential source of random error. In the future, the conclusions of this study need to be validated through multicenter investigations with larger patient cohorts, and future studies will incorporate actual postoperative radiographs for direct methodology comparison. Another limitation is the lack of consideration for varying initial anatomopathological or functional conditions—such as Paprosky classification, Preoperative gait analysis results, number of previous surgeries, implant-bone interface contact area ratio, host bone quality, biomechanical parameters of reconstruction (including center of rotation and offset), and the patient’s overall health status—which may affect surgical outcomes. This omission somewhat compromises the rigor of our discussion on clinical efficacy and screw angulation. In future research, we plan to include these critical variables during the establishment of our case database and perform multivariate analyses. Finally, this study focused exclusively on two biomechanically critical screws, a decision based on our clinical experience and biomechanical analysis. However, this does not imply that other screws make insignificant contributions to the overall stability of the implant. In future research, we intend to develop a biomechanical weighting system, whereby each screw is assigned a weight based on its biomechanical contribution as determined through computational analysis. This approach will enable a more comprehensive evaluation that incorporates all screws, ultimately providing greater clinical value for implant design and surgical decision-making in this field.

## 5 Conclusion

Our 3D evaluation framework for patient-specific hip revision prostheses effectively quantifies critical screw alignment fidelity to preoperative plans, demonstrating strong clinical correlation and reliability. This methodology addresses three fundamental limitations of existing CT-based approaches through enhanced adaptability, defect-robust coordinate systems, and biomechanically focused screw analysis, showing significant potential for clinical translation.

## Data Availability

The original contributions presented in the study are included in the article/supplementary material, further inquiries can be directed to the corresponding authors.
